# The Atlanta College of Physicians and Surgeons

**Published:** 1898-07

**Authors:** 


### THE ATLANTA COLLEGE OF PHYSICIANS AND SURGEONS.

The Atlanta Medical College and the Southern Medical College, both of this city, have been united. For many years both of these schools have been in a very flourishing condition, and naturally both have been rivals for popular prestige. The Atlanta College was the older, and in fact one of the oldest in the South, while on the other hand the Southern was very much younger, yet had already attained a high position for its thorough curriculum. The members of the faculties in the two schools were all good friends, and realizing, as they did, that it would be far better for both schools, and also for the profession in the city and State, could the two schools be united on an equitable basis, conferences looking to the accomplishment of this latter object were instituted, with the result as stated above. The action of the two medical faculties was concurred in by the two boards of trustees, so that the two colleges have been united under the name chosen, "The Atlanta College of Physicians and Surgeons." The new faculty as constituted will be as follows:

A. W. Calhoun, M.D., LL.D., President of the Faculty, Professor of Diseases of the Eye, Ear, Nose and Throat.

J. S. Todd, M.D., Professor of Materia Medica and Therapeutics.

W. S. Kendrick, M.D., Dean of the Faculty, Professor Principles and Practice of Medicine.

W. F. Westmoreland, M.D., Professor of Surgery.

Virgil O. Hardon, M.D., Professor of Obstetrics and Gynecology.

H. P. Cooper, M.D., Professor of Anatomy and Clinical Surgery.

L. H. Jones, A.M., M.D., Professor of Chemistry.

W. S. Elkin, A.B., M.D., Professor of Clinical and Operative Surgery.

W. P. Nicolson, M.D., Professor of Anatomy and Clinical Surgery.

J. C. Johnson, M.D., Professor of Physiology.

F. W. McRae, M.D., Professor of Gastro-intestinal and Rectal Surgery.

Dunbar Roy, A.B., M.D., Clinical Professor of Diseases of the Eye and Ear.

Jno. G. Earnest, M.D., Clinical Professor of Diseases of Women.

